# Supplementary figures and images for: Modeling the effect of daytime duration on the biosynthesis of terpenoid precursors
Source: Front Plant Sci. 2024 Nov 14;15:1465030. doi: 10.3389/fpls.2024.1465030 (PMC11609946; doi:10.3389/fpls.2024.1465030)

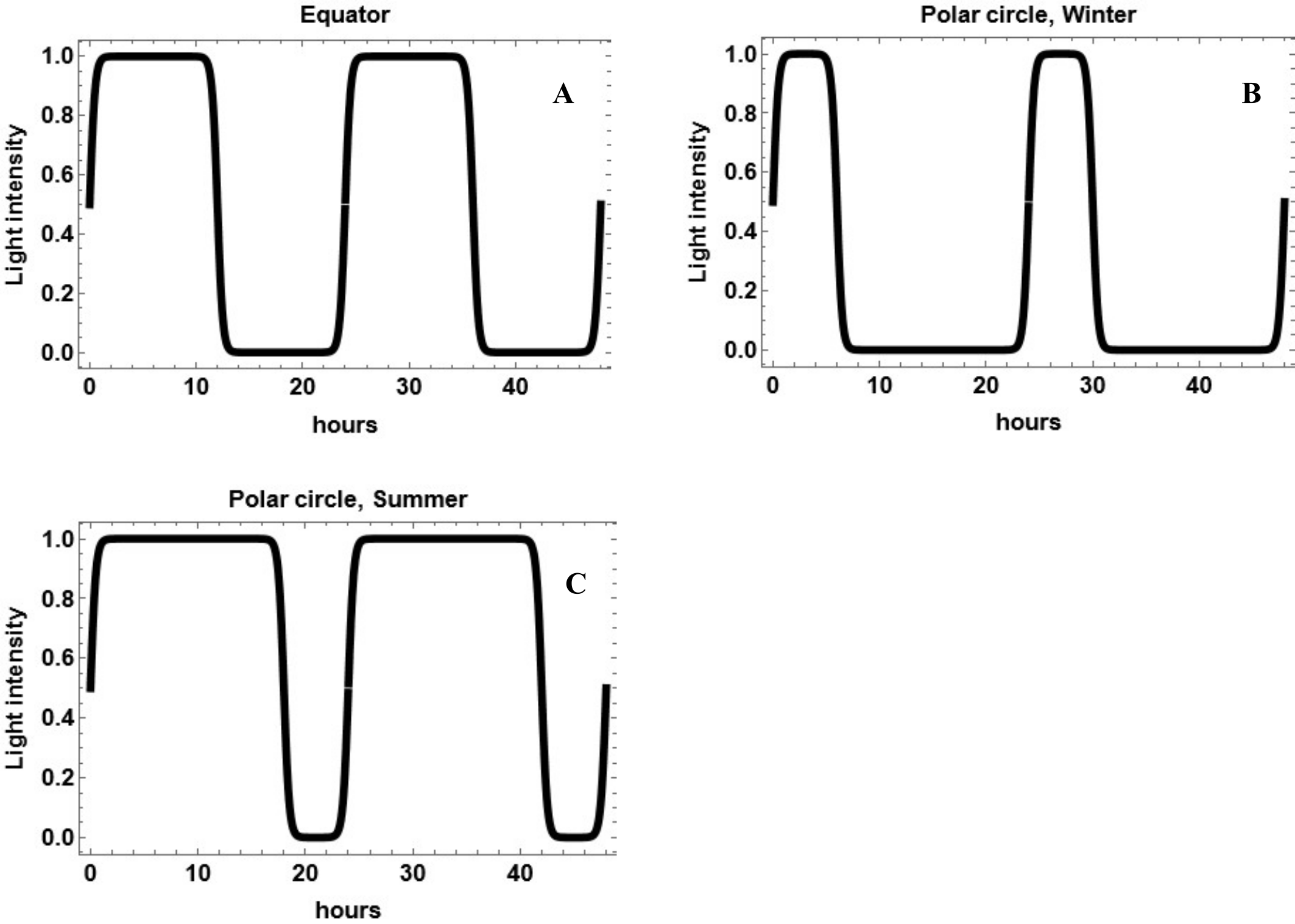

Supplement: Supplementary Figure 1 — Modeling circadian light cycles with the L function. (A) Approximate daylighthours at the equator. (B) Approximate daylighthours at the polar circle during the peak of winter. (C) Approximate daylighthours at the polar circle during the peak of summer. [file Image1.tif]

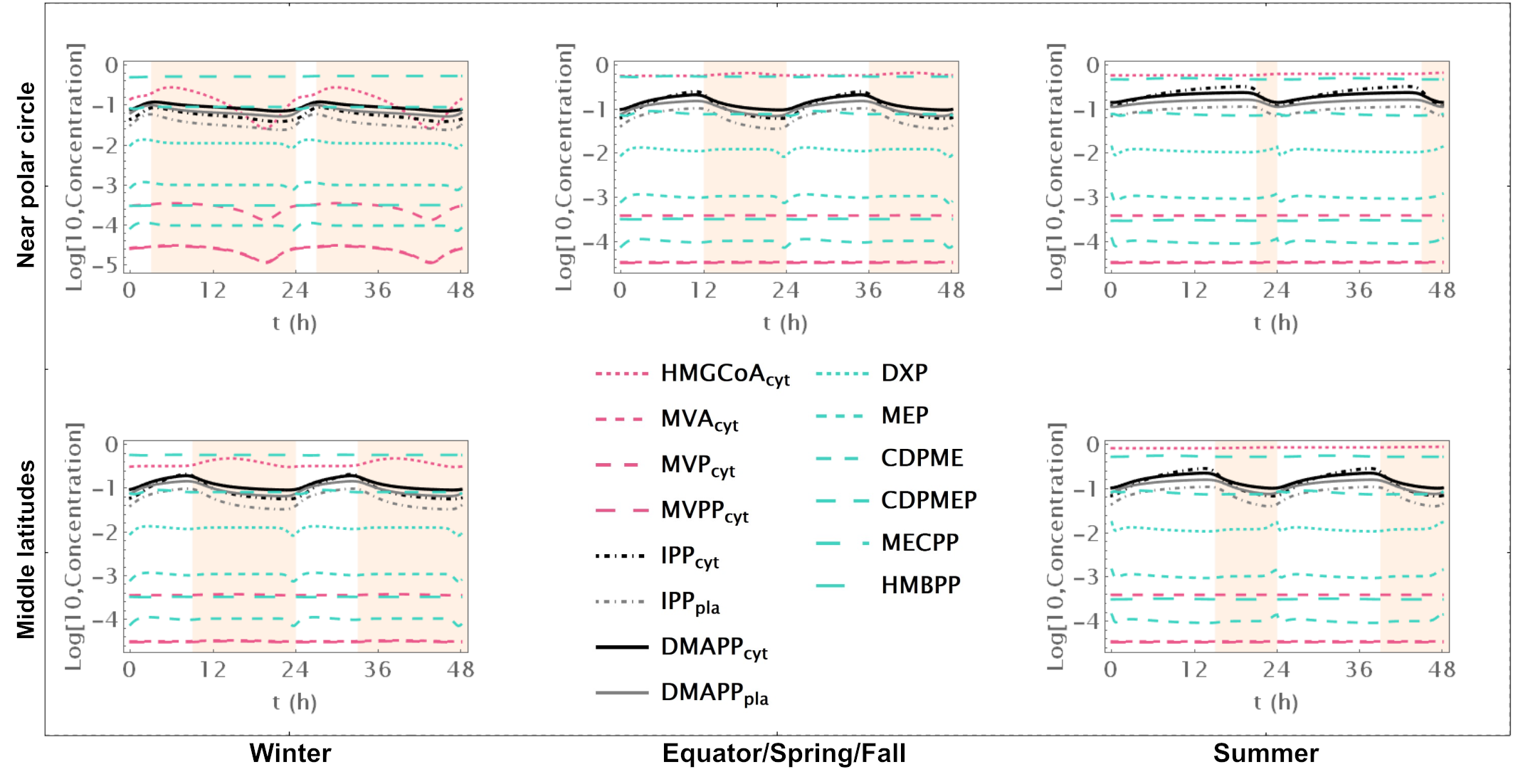

Supplement: Supplementary Figure 2 — Time course simulation of the system throughout 48h at different latitudes and times of the year: equator/spring and fall equinoxes (dusk = 12h), middle latitudes (winter, dusk = 9h; summer, dusk = 15h) and near polar circle latitudes (winter, dusk = 3h; summer, dusk = 21h). T = 1h. [file Image2.tif]

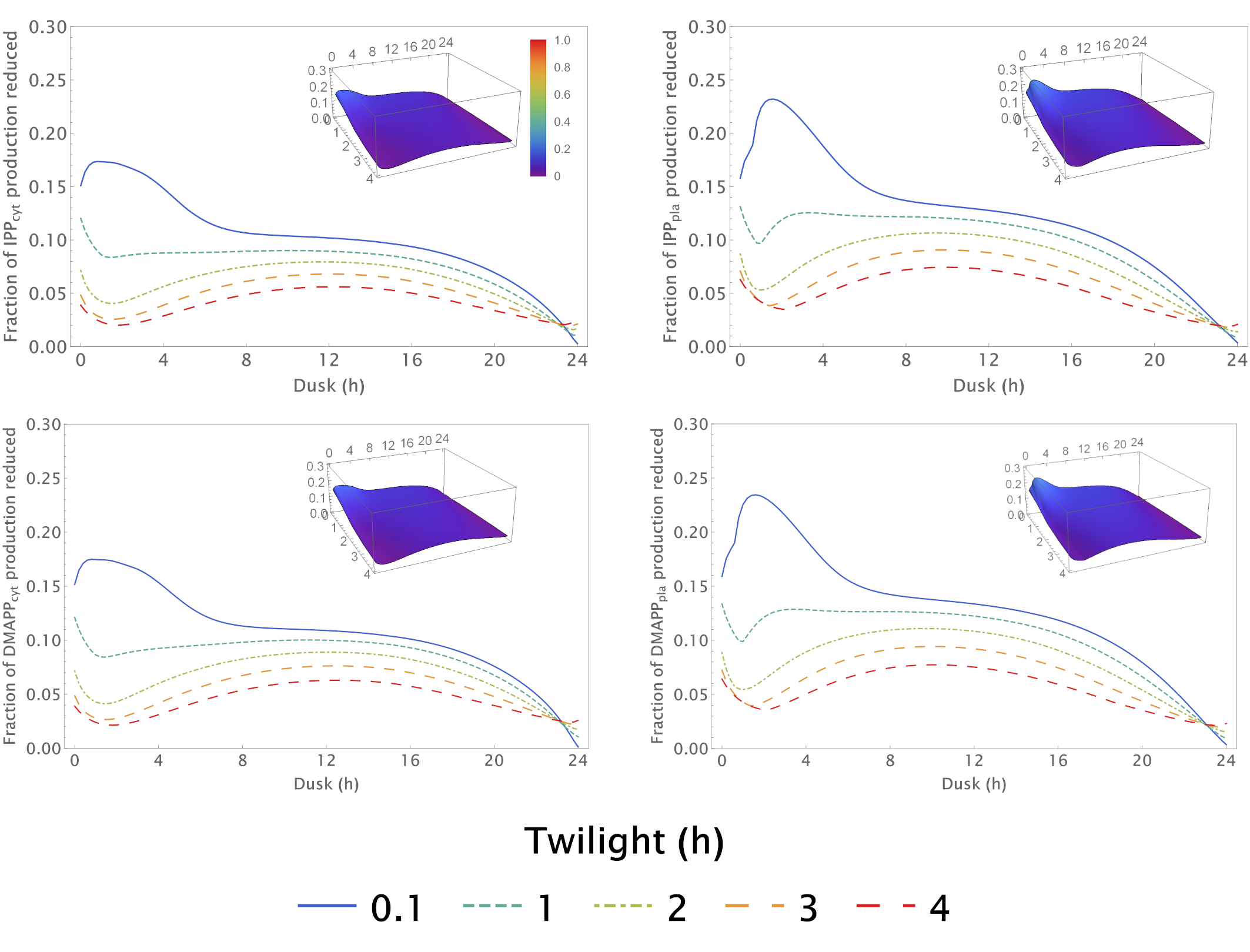

Supplement: Supplementary Figure 3 — Model E. Oscillation amplitude of IPP and DMAPP production (normalized to the maxima) for different values of T (Twilight) and different values of dusk (Daytime). [file Image3.tif]

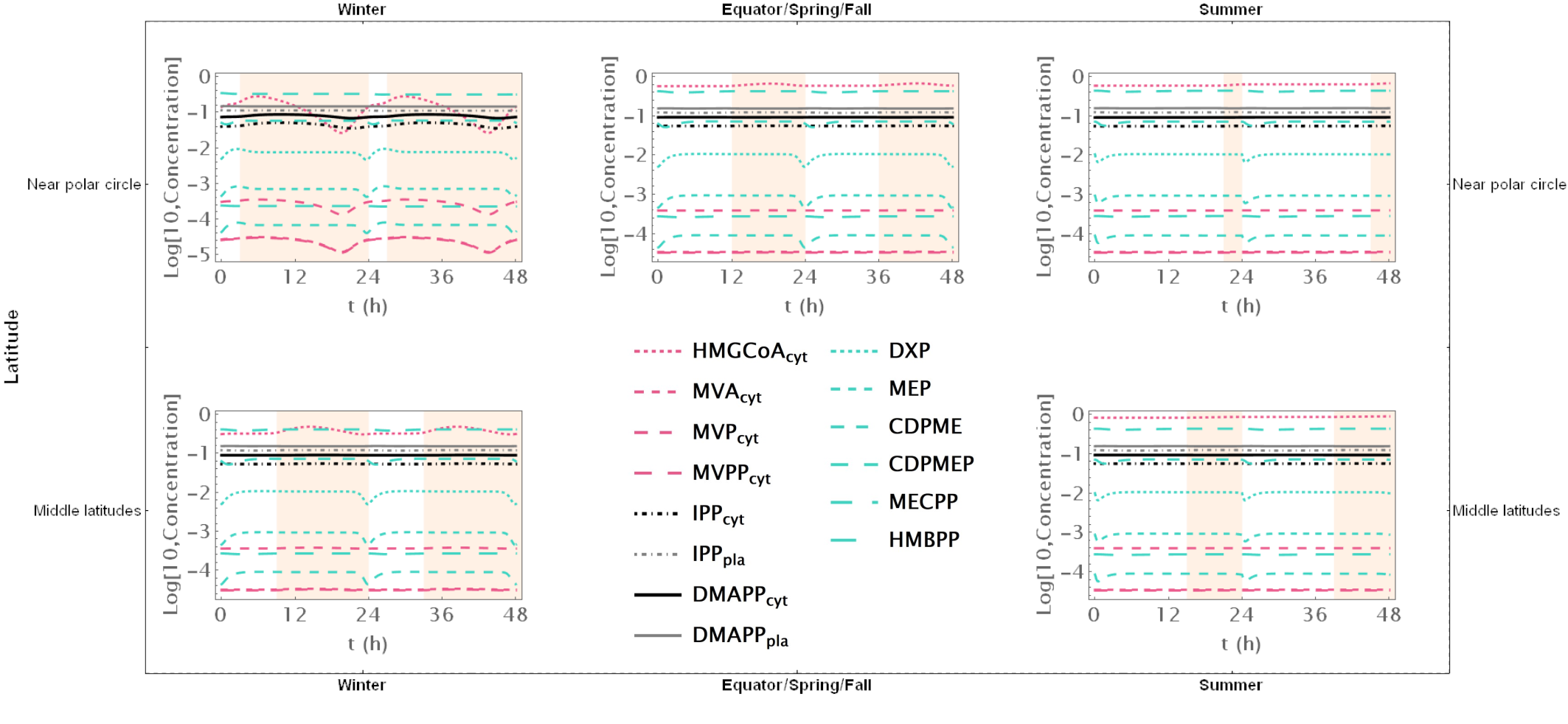

Supplement: Supplementary Figure 4 — Model E without diffusion of IPP/DMAPP between compartments. Time course simulation of the system throughout 48h at different latitudes and times of the year: equator/spring and fall equinoxes (dusk = 12h), middle latitudes (winter, dusk = 9h; summer, dusk = 15h) and near polar circle latitudes (winter, dusk = 3h; summer, dusk = 21h). T = 1h. [file Image4.tif]

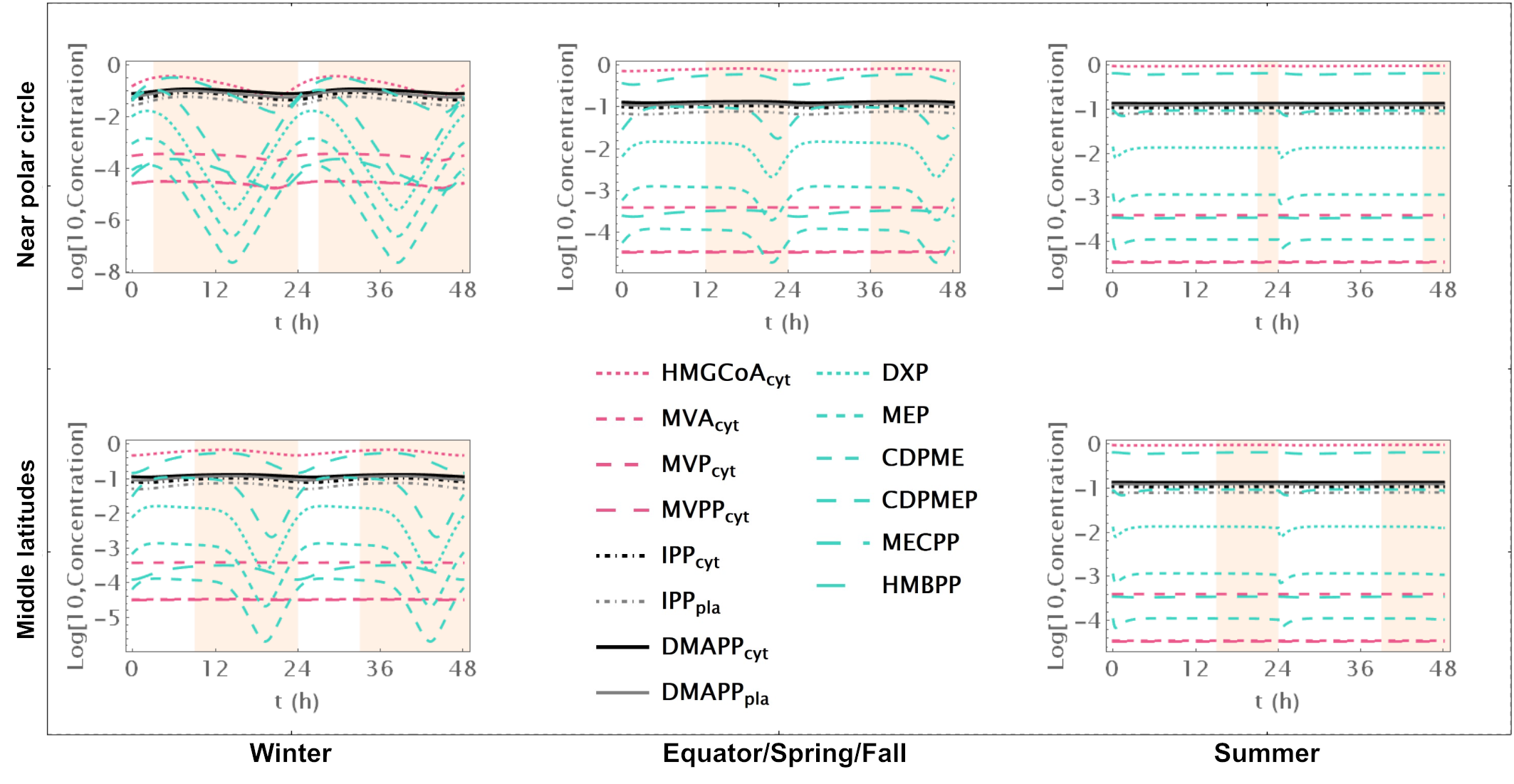

Supplement: Supplementary Figure 5 — Model B. Time course simulation of the system throughout 48h at different latitudes and times of the year: equator/spring and fall equinoxes (dusk = 12h), middle latitudes (winter, dusk = 9h; summer, dusk = 15h) and near polar circle latitudes (winter, dusk = 3h; summer, dusk = 21h). T = 1h. [file Image5.tif]

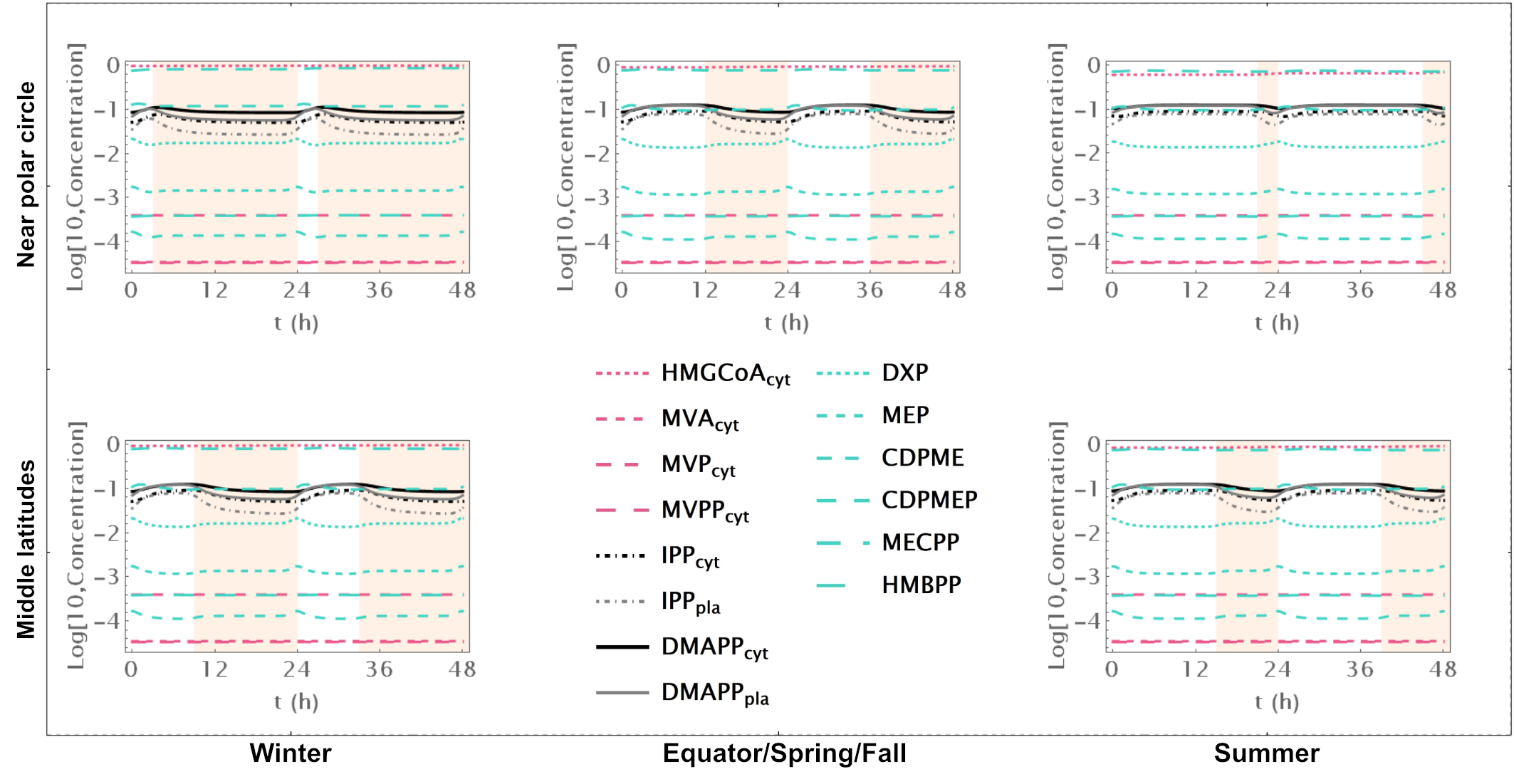

Supplement: Supplementary Figure 6 — Model C. Time course simulation of the system throughout 48h at different latitudes and times of the year: equator/spring and fall equinoxes (dusk = 12h), middle latitudes (winter, dusk = 9h; summer, dusk = 15h) and near polar circle latitudes (winter, dusk = 3h; summer, dusk = 21h). T = 1h. [file Image6.tif]

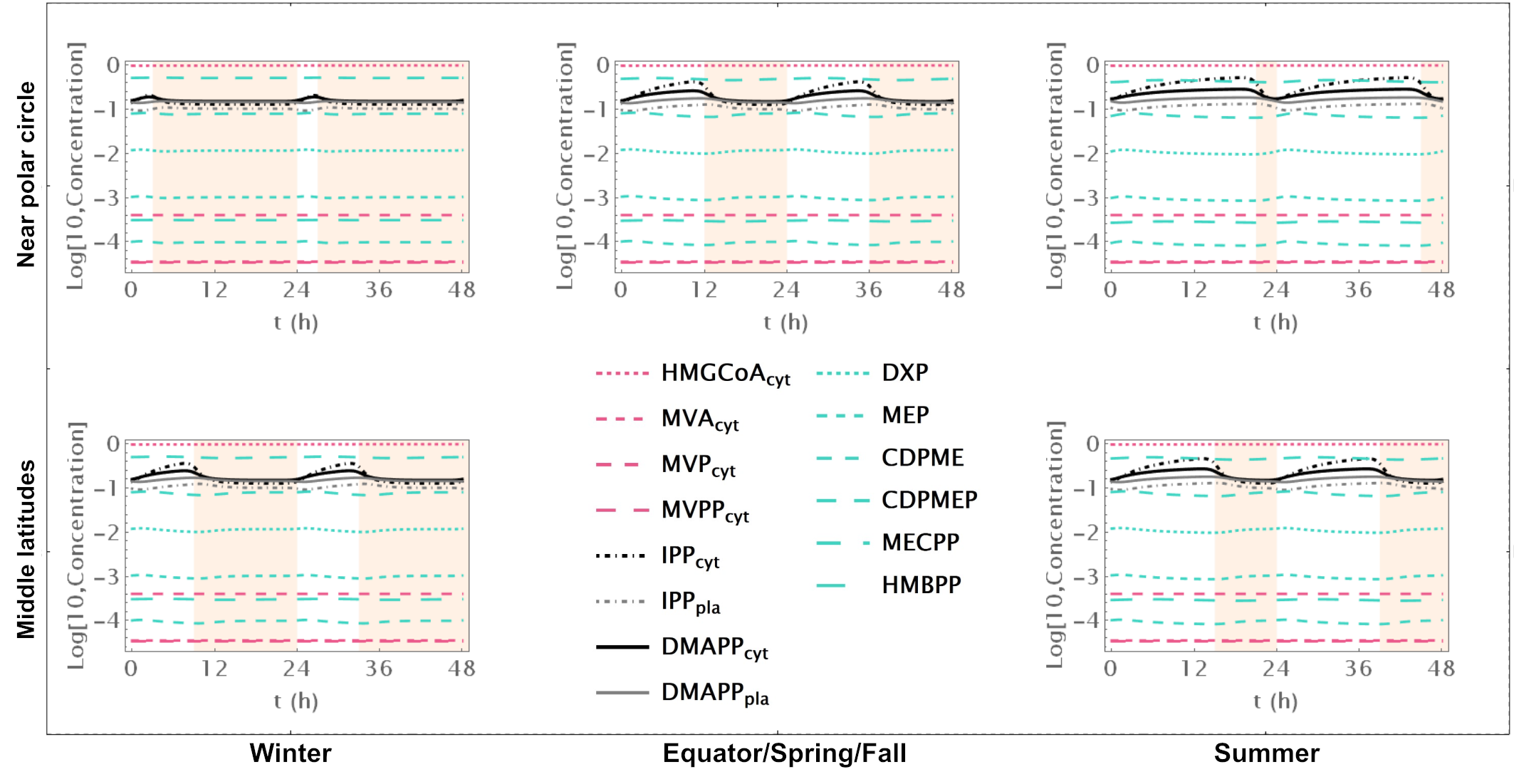

Supplement: Supplementary Figure 7 — Model D. Time course simulation of the system throughout 48h at different latitudes and times of the year: equator/spring and fall equinoxes (dusk = 12h), middle latitudes (winter, dusk = 9h; summer, dusk = 15h) and near polar circle latitudes (winter, dusk = 3h; summer, dusk = 21h). T = 1h. [file Image7.tif]
